# Renal sympathetic denervation restores aortic distensibility in patients with resistant hypertension: data from a multi-center trial

**DOI:** 10.1007/s00392-018-1229-z

**Published:** 2018-03-08

**Authors:** Lukas Stoiber, Felix Mahfoud, Seyedeh Mahsa Zamani, Tomas Lapinskas, Michael Böhm, Sebastian Ewen, Saarraaken Kulenthiran, Markus P. Schlaich, Murray D. Esler, Tommy Hammer, Knut Haakon Stensæth, Burkert Pieske, Stephan Dreysse, Eckart Fleck, Titus Kühne, Marcus Kelm, Philipp Stawowy, Sebastian Kelle

**Affiliations:** 1 Department of Internal Medicine/Cardiology, German Heart Center Berlin, Augustenburger Platz 1, 13353 Berlin, Germany; 2 Division of Cardiology, Angiology and Intensive Internal Medicine, Department of Internal Medicine III, Saarland University, Homburg, Germany; 3 Institute for Medical Engineering and Science, Massachusetts Institute of Technology, Cambridge, MA USA; 4 Dobney Hypertension Centre, School of Medicine, University of Western Australia-Royal Perth Hospital Unit, Crawley, Australia; 5 Baker Heart and Diabetes Institute, Melbourne, Australia; 6 Department of Radiology and Nuclear Medicine, St. Olavs Hospital and Institute of Circulation and Medical Imaging, Norwegian University of Science and Technology, Trondheim, Norway; 7 Department of Internal Medicine/Cardiology, Charité Campus Virchow Klinikum, Berlin, Germany; 8 Department of Cardiology, Medical Academy, Lithuanian University of Health Sciences, Kaunas, Lithuania; 9 DZHK (German Center for Cardiovascular Research), Partner Site Berlin, Berlin, Germany; 10 Department of Congenital Heart Disease and Pediatric Cardiology, German Heart Center Berlin, Berlin, Germany; 11 Institute for Computational and Imaging Science in Cardiovascular Medicine, Charité Berlin, Berlin, Germany

**Keywords:** Renal denervation, Aortic distensibility, Compliance, Vascular stiffness, Cardiovascular magnetic resonance, CMR, Resistant hypertension

## Abstract

Renal sympathetic denervation (RDN) is under investigation as a treatment option in patients with resistant hypertension (RH). Determinants of arterial compliance may, however, help to predict the BP response to therapy. Aortic distensibility (AD) is a well-established parameter of aortic stiffness and can reliably be obtained by CMR. This analysis sought to investigate the effects of RDN on AD and to assess the predictive value of pre-treatment AD for BP changes. We analyzed data of 65 patients with RH included in a multicenter trial. RDN was performed in all participants. A standardized CMR protocol was utilized at baseline and at 6-month follow-up. AD was determined as the change in cross-sectional aortic area per unit change in BP. Office BP decreased significantly from 173/92 ± 24/16 mmHg at baseline to 151/85 ± 24/17 mmHg (*p* < 0.001) 6 months after RDN. Maximum aortic areas increased from 604.7 ± 157.7 to 621.1 ± 157.3 mm^2^ (*p* = 0.011). AD improved significantly by 33% from 1.52 ± 0.82 to 2.02 ± 0.93 × 10^−3^ mmHg^−1^ (*p* < 0.001). Increase of AD at follow-up was significantly more pronounced in younger patients (*p* = 0.005) and responders to RDN (*p* = 0.002). Patients with high-baseline AD were significantly younger (61.4 ± 10.1 vs. 67.1 ± 8.4 years, *p* = 0.022). However, there was no significant correlation of baseline AD to response to RDN. AD is improved after RDN across all age groups. Importantly, these improvements appear to be unrelated to observed BP changes, suggesting that RDN may have direct effects on the central vasculature.

**Electronic supplementary material:**

The online version of this article (10.1007/s00392-018-1229-z) contains supplementary material, which is available to authorized users.

## Introduction

Arterial stiffness elevates systolic blood pressure (SBP) as it accelerates pressure waves returning to the heart [1]. Patients with resistant hypertension (RH), defined as uncontrolled hypertension despite the concurrent use of at least three antihypertensive drugs including a diuretic, are at high risk for cardiovascular events [2, 3]. Aortic distensibility (AD) provides an estimation of the elastic response to the pulsatile blood flow and is regarded as one of the key parameters of aortic elasticity. AD measurements are worthwhile to detect even subclinical vascular changes resulting in response to aging and increased systolic pressure [4, 5]. While advanced age seems to be the major factor of the arterial remodeling process, decrease in AD is more pronounced before the fifth decade of life [1, 5]. Cardiac magnetic resonance imaging (CMR) allows non-invasive imaging of the entire aorta and makes evaluation of AD accessible and accurate [6, 7].

Catheter-based renal denervation (RDN) has been suggested as a treatment option for RH, aiming at modulating renal and central sympathetic activity as one of the factors contributing to elevated BP, and likely increased arterial stiffness and thus afterload. RDN has also been shown to reduce target organ damage, in particular left ventricular hypertrophy, an effect that seemed to be independent of its blood-pressure lowering capacity [3, 8, 9].

However, the results of the latest randomized trials on RDN were mixed and considerable variability in the response to RDN was observed among patients [10–13]. The current study aimed to assess the effects of RDN on aortic structural and functional characteristics and to investigate whether pre-treatment AD can predict the BP response to RDN.

## Methods

### Study population and design

This present trial was planned as a prospective, multi-center trial and implemented at four investigational sites: two in Germany, one in Norway, and one in Australia. A total of 65 patients with RH undergoing RDN between May 2009 and January 2014 were prospectively enrolled. RH was defined as an office SBP above goal (≥ 140 mmHg) or mean ambulatory 24-h SBP > 135 mmHg despite the use of ≥ 3 antihypertensive agents of different classes at maximum or highest tolerated doses, including a diuretic [2, 14]. BP measurement methods are described in detail elsewhere [14–16]. Office BP was obtained at entry and 6 months after treatment. Office BP readings were taken during the MRI with an automatic brachial oscillometric Omron HEM-705 monitor (Omron Healthcare, Vernon Hills, IL) after at least 5 min of rest according to the Standard Joint National Committee VII Guidelines. Averages of the triplicate measures were calculated and AD was calculated from the averaged brachial PP. Patients with general contraindications for CMR were excluded as well as patients with contraindications for RDN [15]. A stable antihypertensive drug regimen was another inclusion criterion and changes in treatment during the study period were only permitted when medically required. A standardized CMR protocol was followed at both baseline and 6-month follow-up to assess myocardial function and volumes. BP was determined during both MR examinations to quantify AD. Clinical assessment, including history taking and physical examination, evaluation of vital signs, and review of medication compliance, was performed at both timepoints. A Symplicity Flex system catheter (Medtronic, Minneapolis, MN, USA) was used in the RDN procedures. The study was approved by the local institutional review board (Charité-Universitätsmedizin Berlin) in accordance with all the ethical standards and written informed consent was provided by all patients before inclusion. Measurements were performed as an extension to the protocols of the Symplicity trials (NCT00664638, NCT00888433, and NCT01888315). The results presented here are an extension to the work previously published, based on 55 patients of the same cohort [3].

### CMR protocol

All studies were performed before and 6 months after RDN using a 1.5 T Achieva MRI scanner (Philips Healthcare, Best, The Netherlands) or 1.5 T Siemens Symphony or a 1.5 T Siemens Aera MRI system (Siemens Healthcare Sector, Erlangen, Germany). Cine images were acquired during breath holds of 10–15 s using vector electrocardiogram gating and steady-state free precession sequence (SSFP).

### CMR analysis

#### LV measurements

CMR measurements were performed as previously reported and in accordance with the recommendations of the task force for post-processing of the Society for Cardiovascular MR [17, 18]. We used Qmass software version 8.1 (Medis Suite version 2.1., Medis, The Netherlands) for offline CMR analyses. Endocardial and epicardial borders were traced manually at end-diastole and end-systole, with exclusion of the papillary muscles from LVM to achieve better reproducibility [19]. LV volumes and mass were calculated using the summation of slices method [20]. LV measurements including wall thickness and internal dimensions were obtained using the SAX view basal to the tips of the papillary muscles [21].

#### Aortic area measurements

The inner diameter of the aortic wall was traced manually and contouring was then adapted with the contouring tool in Qmass software version 8.1 (Medis Suite version 2.1., Medis, The Netherlands). All measures were performed three times and then averaged. We used cross-sectional areas of the descending aorta obtained in the standard 4-chamber cine images at baseline and 6-month follow-up. For the evaluation of intra- and inter-observer variability, aortic area measurements were repeated by both the first observer and a second observer in ten patients.

To calculate AD, we first determined aortic strain, defined as the relative change in area, and then normalized this value with the peripheral PP obtained at the time point of the CMR (average of three measures). This relation can, as previously published, be described as

$${\text{Aortic distensibility}}~=\frac{{{A_{{\text{max}}}} - {A_{{\text{min}}}}}}{{{A_{{\text{min}}}} \times {\text{pulse pressure}}}},$$

where *A*_max_ and *A*_min_ refer to the corresponding maximal and minimal cross-sectional areas of the descending aorta in our case [5, 22].

### Statistical analysis

All data are presented as mean ± standard deviation. Differences in mean values were compared using Student’s *T* test if data were normally distributed or the Wilcoxon test if normality could not be assumed. Kolmogorov–Smirnov test was used to assess distribution. Mann–Whitey-*U* test was used to compare responders with non-responders. To compare the characteristics between different groups regarding AD, we used ANOVA and Kruskal–Wallis tests whenever variables were continuous and the Chi-square test or Fisher’s exact test for categorical variables. To address the regression to the mean phenomenon and the confounding by indication issue, we used general linear models (GLM) for repeated measurements including covariates. We assessed the effect of RDN between certain groups as previously described using baseline SBP and DBP as covariates in the calculation [23]. Univariate correlations between parameters were obtained using Pearson’s correlation coefficients. Intra- and inter-observer variability is displayed in Bland–Altman plots in our supplementary material. In addition, the intra-class correlation coefficient (ICC) was considered as excellent with a value of > 0.7 [24]. A *p* value < 0.05 was considered statistically significant. All statistical analyses were performed using IBM SPSS Statistics for Windows (Version 24.0, SPSS Inc., Chicago, IL, USA).

#### Reproducibility

Bland–Altman plots and ICC analysis are provided in Supplemental Figure S2 and Supplemental Table S2 for intra-observer and inter-observer variability measurements for cross-sectional aortic area. The intra- and inter-observer concordances (95% confidence interval) were 0.940 (0.886–0.968) and 0.993 (0.987–0.996), respectively, indicating excellent consistency of repeated evaluations.

## Results

### Study population

Sixty-five patients with RH were included in this analysis. Seven patients had to be excluded due to low image quality. RDN was performed successfully in all patients. No loss to follow-up was reported during the study period of 6 months. The baseline clinical characteristics of the patients included in the analysis are presented in Table 1.

**Table 1:** Baseline characteristics of the study cohort

Parameter	All patients (*n* = 58)
Baseline clinical characteristics of the study cohort	
Age (years)	64.4 ± 9.6
Male	42 (72%)
BMI (kg m^−2^)	29.3 ± 4.2
Stroke	8 (14%)
Type 2 diabetes	26 (45%)
No. of antihypertensive drugs	4.6 ± 1.6
ACE inhibitors/ARBs	51 (88%)
β-Blockers	49 (84%)
Calcium channel blockers	45 (78%)
Diuretics	46 (79%)
Sympatholytics	24 (41%)
Direct renin inhibitors	18 (31%)
No. of patients with isolated SHT	28 (48%)
Baseline hemodynamics of the study cohort	
Systolic BP (mmHg)	172.8 ± 23.6
Diastolic BP (mmHg)	92.3 ± 16.1
Pulse pressure (mmHg)	79.6 ± 15.5

Data are expressed as mean and standard deviation

*No*. number, *BMI* body mass index, *SHT* systolic hypertension, *BP* blood pressure, *ACE* angiotensin converting enzyme, *ARB* angiotensin receptor blocker

### Blood pressure

Office systolic and diastolic BP decreased significantly from 173/92 ± 24/16 mmHg at baseline to 151/85 ± 24/17 mmHg (*p* < 0.001) 6 months after RDN.

### LV measurements

Functional and anatomical parameters are depicted in Table 2. No significant changes between baseline and 6 months were observed for normalized LV end-diastolic volume (LVEDVI 85 ± 22 vs. 84 ± 23 mL m^−2^; *p* = 0.325); however, normalized LV end-systolic volume (LVESVI 39 ± 17 vs. 36 ± 15 mL m^−2^; *p* = 0.045) decreased significantly. There were no significant changes of LV internal dimensions at 6 months. Ejection fraction increased from 55.4 ± 11 to 57.5 ± 9.3%, *p* = 0.057. LV mass indexed to BSA (LVMI) significantly decreased from 57.7 ± 16.3 to 54.4 ± 15.4 g m^−2^, *p* < 0.001.

**Table 2:** Patients’ anatomic, hemodynamic, and arterial measures at baseline and follow-up

Parameter	Baseline	6-month follow-up	*p* value
Anatomic and functional analysis (*n* = 50 patients)			
LVEDVI (mL m^−2^)	85.0 ± 21.9	83.9 ± 22.4	0.325
LVESVI (mL m^−2^)	38.6 ± 16.5	36.0 ± 14.9	0.045
IVSTd (mm)	12.3 ± 3.6	12.0 ± 3.3	0.262
LVIDd (mm)	56.7 ± 6.4	56.6 ± 6.4	0.939
LV mass/BSA (g m^−2^)	57.7 ± 16.3	54.4 ± 15.4	< 0.001
LA size (cm^2^)	25.6 ± 7.2	25.2 ± 6.4	0.257
Global circumferential strain (%)	− 20.7 ± 7.3	− 21.2 ± 7.1	0.280
LVEF (%)	55.4 ± 11.0	57.5 ± 9.3	0.057
Hemodynamics (*n* = 58 patients)			
Systolic BP (mmHg)	172.8 ± 23.6	151.4 ± 24.2	< 0.001
Diastolic BP (mmHg)	92.3 ± 16.1	84.6 ± 16.5	< 0.001
Pulse pressure (mmHg)	80.5 ± 15.0	66.9 ± 16.6	< 0.001
Cross-sectional areas descending aorta (mm^2^) (*n* = 58 patients)			
Maximum area (ES)	604.7 ± 157.7	621.1 ± 157.3	0.011
Minimal area (ED)	541.5 ± 138.5	553.6 ± 155.2	0.110
Aortic area change absolute	63.2 ± 34.3	67.5 ± 25.4	0.153
Aortic area change % (aortic strain)	11.7 ± 5.6	12.8 ± 5.5	0.262
Descending aortic distensibility (10^−3^ mmHg^−1^)	1.52 ± 0.82 1.47 (0.90)^#^	2.02 ± 0.93 1.77 (1.02)^#^	< 0.001 < 0.001

Data are expressed as mean and standard deviation

*LVEDVI* left ventricular end-diastolic volume index, *LVESVI* left ventricular end-systolic volume index, *IVSTd* interventricular septal thickness at diastole, *LVIDd* left ventricular internal diameter at diastole, *BSA* body surface area, *LV* left ventricle, *LA* left atrium, *EF* ejection fraction, *BP* blood pressure, *ES* end-systolic, *ED* end-diastolic

^#^For AD, values are also given as median (interquartile range). All *p* values are from the Wilcoxon test

### Arterial measurements

Arterial measurements included data of minimal and maximal cross-sectional areas of the descending aorta and absolute changes in aortic areas as well as aortic strain. Data are available for all patients (*n* = 58) and are summarized in Table 2. Maximum aortic area increased significantly from 604.7 ± 157.7 mm^2^ at baseline to 621.1 ± 157.3 mm^2^ (*p* = 0.011) 6 months after RDN. A non-significant trend was observed for an increase of minimal aortic area, absolute change in aortic area, and aortic strain between baseline and 6-month follow-up.

### Distensibility measurements

**Fig. 1:**
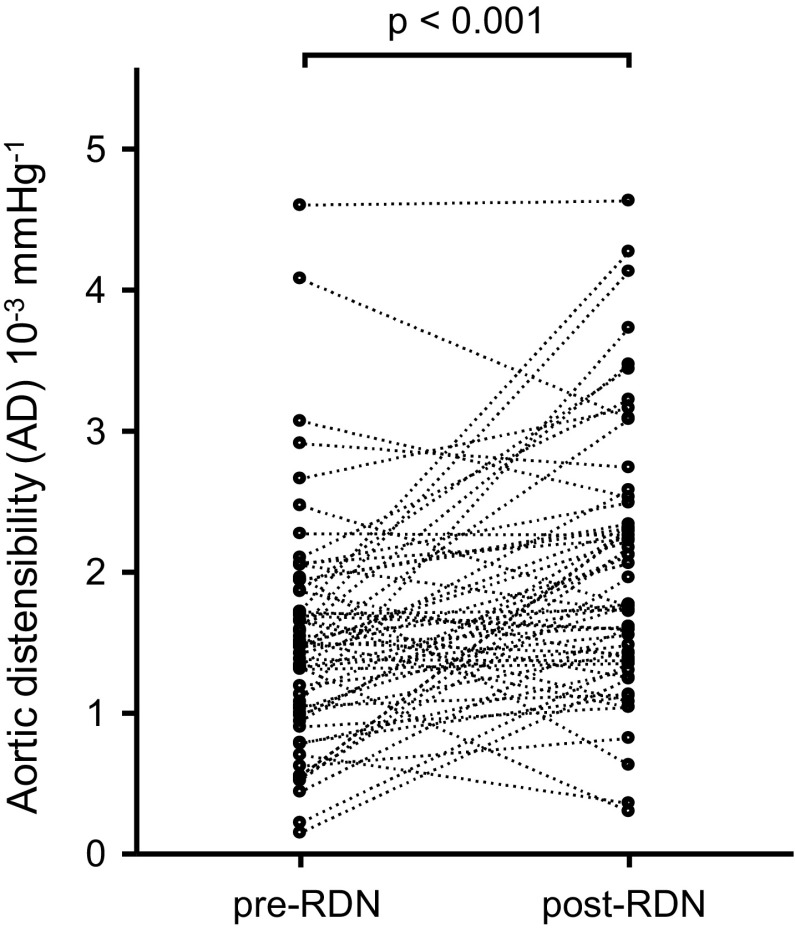
Distribution of aortic distensibility (AD) at baseline and 6-month follow-up. AD had increased by 33% post RDN at 6-month follow-up

Values of distensibility were based on the aortic strain and pulse pressure obtained. In general, AD increased significantly by 33% from 1.52 ± 0.82 × 10^−3^ mmHg^−1^ at baseline to 2.02 ± 0.93 × 10^−3^ mmHg^−1^ at follow-up (*p* < 0.001) (Fig. 1).

### Age-related changes in distensibility

**Fig. 2:**
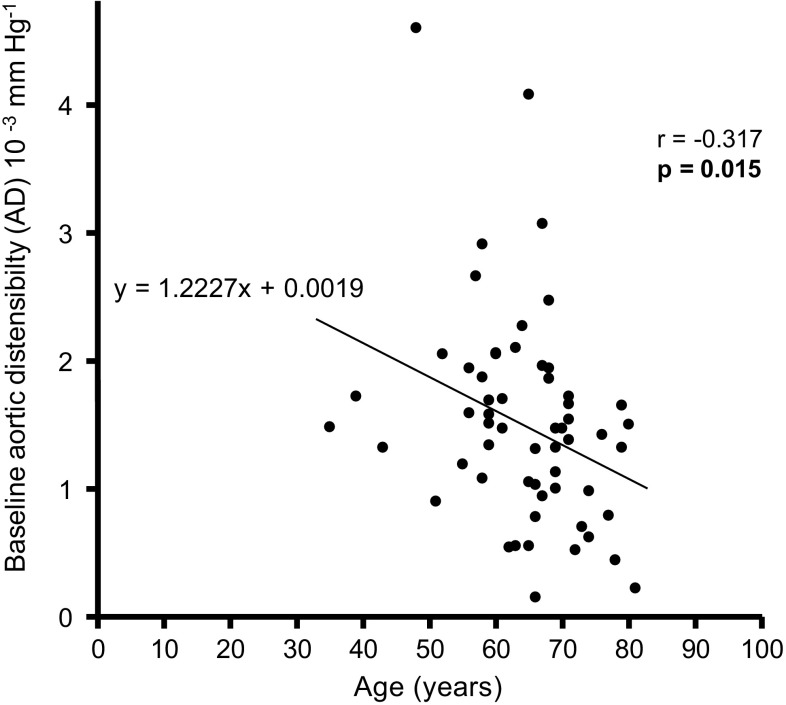
Correlations of baseline aortic distensibility (AD) and patients age

**Fig. 3:**
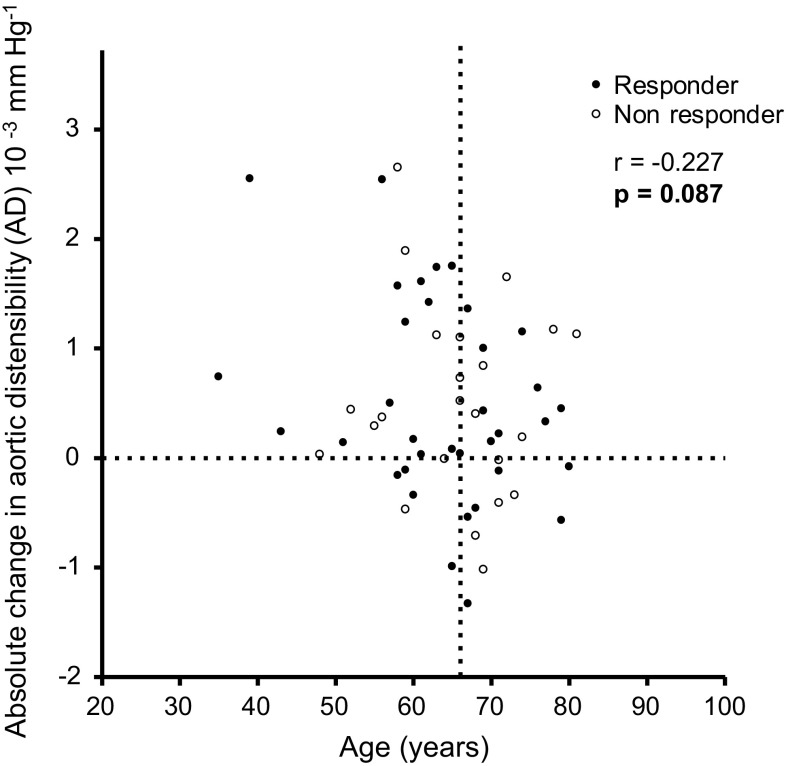
Correlations of absolute change in aortic distensibility (AD) at 6-month follow-up and patients age. Response to RDN was defined as a ≥ 10 mmHg decrease in SBP

**Fig. 4:**
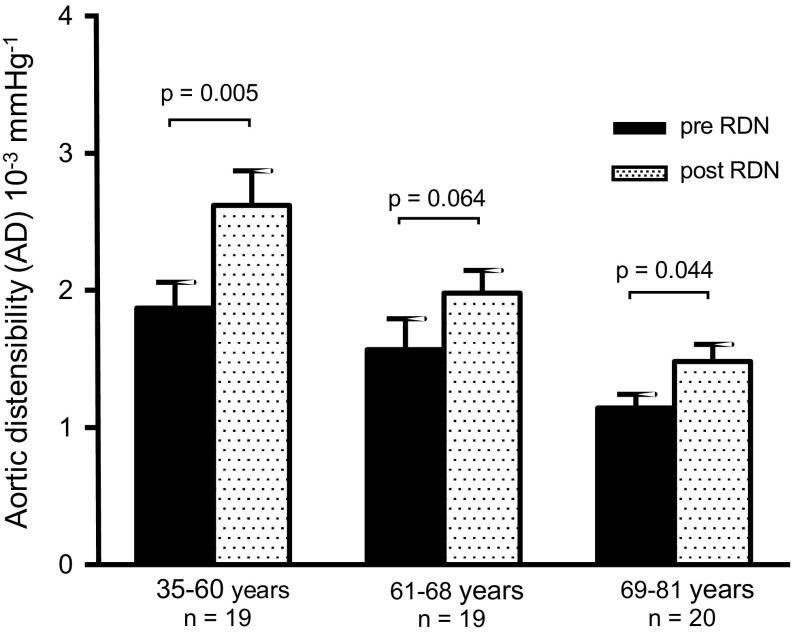
Changes in aortic distensibility (AD) from baseline to 6-month follow-up regarding age group at baseline

The baseline AD was age-dependent (*p* = 0.015) and the increase in AD was more pronounced in younger patients (Figs. 2, 3). Pearson’s estimates showed a non-significant correlation between age and change in AD (*p* = 0.087) (Table S1). To further evaluate the impact of age on AD, patients were separated into tertiles related to age at baseline (Table 3). The group with young age (35–60 years) showed the highest change in AD, from 1.87 ± 0.82 × 10^−3^ mmHg^−1^ at baseline to 2.62 ± 1.10 × 10^−3^ mmHg^−1^ at follow-up (*p* = 0.005). Interestingly, AD increased as well in the moderate age subgroup (61–68 years), from 1.57 ± 0.98 to 1.98 ± 0.72 × 10^−3^ mmHg^−1^ (*p* = 0.064), and even in the highest age subgroup (69–81 years), from 1.14 ± 0.45 to 1.48 ± 0.56 × 10^−3^ mmHg^−1^ (*p* = 0.044), indicating that RDN improved arterial stiffness also in the elderly study population (Fig. 4). Baseline adjusted changes in AD between these three groups differed significantly (*p* = 0.008).

**Table 3:** Aortic distensibility for the entire cohort by tertile of age at baseline

Parameter	1st age tertile 35–60 years (*n* = 19)	2nd age tertile 61–68 years (*n* = 19)	3rd age tertile 69–81 years (*n* = 20)	*p* value
Pulse pressure (mmHg)				
Baseline	78.4 ± 14.7	78.9 ± 16.8	81.3 ± 15.6	0.826
6-month follow-up	59.8 ± 14.2	68.1 ± 11.9	67.4 ± 17.6	0.166
Descending aortic distensibility (10^−3^ mmHg^−1^)				
Baseline	1.87 ± 0.82	1.57 ± 0.98	1.14 ± 0.45	0.019
6-month follow-up	2.62 ± 1.10	1.98 ± 0.72	1.48 ± 0.56	< 0.001
Absolute change at 6-month follow-up	0.75 ± 1.01	0.41 ± 0.96	0.34 ± 0.68	0.318/0.008*
Relative change at 6-month follow-up	40 ± 54%	26 ± 61%	29 ± 30%	0.459/0.434**

*p* values by ANOVA

*Univariable Bonferroni correction using baseline distensibility as covariable

**1st vs. 2nd and 1st vs. 3rd tertile

### Distensibility and response to RDN

In total, 37/58 (64%) patients after RDN showed an SBP reduction of at least 10 mmHg and were subsequently defined as “responders” [25]. Differences in results between responders and non-responders are summarized in Table 4. Absolute AD values after RDN increased in all patients regardless of the treatment response. AD increased from 1.51 ± 0.8 to 2.0 ± 0.84 × 10^−3^ mmHg^−1^ (*p* = 0.002) in responders and from 1.54 ± 0.93 to 1.99 ± 1.11 × 10^−3^ mmHg^−1^ (*p* = 0.046) in non-responders (Figure S1). Interestingly, in 18/21 (86%) non-responders, maximum aortic areas significantly increased from 648.2 ± 192.6 to 675.4 ± 201.7 mm^2^ (*p* = 0.005), whereas this change was less pronounced in responders, with 20/37 (54%) increasing from 580.0 ± 130.7 to 590.3 ± 117.7 mm^2^ (*p* = 0.294).

**Table 4:** Cross-sectional aortic area measurements and aortic distensibility: comparison of baseline and follow-up parameters in patients with and without response to renal sympathetic denervation

Parameter	Responders (*n* = 37)			Non-responders (*n* = 21)			*p* value	*p* value
	Baseline	Follow-up	*p* value	Baseline	Follow-up	*p* value	Baseline Responder vs. non	Follow-up Responder vs. non
Cross-sectional area descending aorta (mm^2^)								
Maximum area	580.0 ± 130.7	590.3 ± 117.7	0.294	648.2 ± 192.6	675.4 ± 201.7	0.005	0.216	0.137*
Minimal area	516.6 ± 109.5	525.7 ± 109.5	0.346	585.4 ± 173.0	602.8 ± 207.6	0.140	0.143	0.541*
Aortic area change	63.4 ± 36.4	64.5 ± 22.7	0.582	62.9 ± 30.9	72.6 ± 29.4	0.122	0.680	0.258*
Aortic area change % (aortic strain)	12.2 ± 6.0	12.6 ± 4.9	0.746	10.8 ± 4.9	13.2 ± 6.5	0.144	0.668	0.336*
Area change absolute	1.1 ± 29.0			9.7 ± 34.4			0.336	
Distensibility descending aorta (10^−3^ mmHg^−1^)								
Absolute values	1.51 ± 0.8	2.0 ± 0.84	0.002	1.54 ± 0.93	1.98 ± 1.11	0.046	0.942	0.737*
Distensibility change absolute	0.53 ± 0.91			0.44 ± 0.89			0.692 (0.686**)	
Distensibility change %	35.1 ± 60.1			28.6 ± 58.0			0.6901	
Pulse pressure (mmHg)								
Absolute values	82.9 ± 14.2	63.7 ± 12.1	< 0.001	73.8 ± 16.2	67.6 ± 19.2	0.265	0.031	0.346

Response defined as systolic blood-pressure reduction at 6-month follow-up ≥ 10 mmHg. Data are expressed as mean and standard deviation

*BL adjusted univariable analyses with Bonferroni estimates

**Age and baseline distensibility as covariables

### Distensibility amount groups

Based on the median AD at baseline, patients were divided into two groups. An absolute AD value of ≥ 1.4747 × 10^−3^ mmHg^−1^ was considered high-baseline AD. Comparison of baseline characteristics with respect to AD is summarized in Table 5. High-baseline AD patients were significantly younger than the low-baseline AD patients (61.4 ± 10.1 vs. 67.1 ± 8.4 years, *p* = 0.022). No difference was observed in the other baseline clinical parameters. Office systolic and diastolic BP decreased significantly from 168/93 ± 22/15 mmHg at baseline to 149/86 ± 22/17 mmHg (*p* < 0.001) 6 months after RDN in the high AD group and from 177/92 ± 25/17 mmHg at baseline to 153/83 ± 26/16 mmHg (*p* < 0.001) in the low-baseline AD group; the difference in change between the groups was 6/2 mmHg.

**Table 5:** Patients’ clinical characteristics according to amount of aortic distensibility at baseline and hemodynamic characteristics at baseline and follow-up

Clinical parameter	Low distensibility 0.15–1.47 (10^−3^ mmHg^−1^) *n* = 30				High distensibility 1.48–4.6 mmHg^−1^ *n* = 28				*p* value^2,4^	
Age (years)	67.1 ± 8.4				61.4 ± 10.1				0.022	
Male	21 (70%)				21 (75%)				0.670	
BMI (kg m^−2^)	29.5 ± 3.7				29.2 ± 4.8				0.830	
Stroke	3 (10%)				5 (18%)				0.464	
Type 2 diabetes	14 (47%)				12 (43%)				0.798	
Nb. of BP drugs	4.9 ± 1.7				4.3 ± 1.6				0.154	
More than 3 BP drugs	23 (77%)				22 (78%)				1.000	

Hemodynamic parameters	Low distensibility			*p* value^1^	High distensibility			*p* value^1^	*p* value^2^	*p* value^3^
	Baseline	Follow-up	∆ (95% CI)		Baseline	Follow-up	∆ (95% CI)		Baseline High vs. low	Follow-up High vs. low
Systolic BP (mmHg)	177.3 ± 24.6	153.4 ± 26.4	− 24.0 ± 26.5	0.001	167.9 ± 22.0	149.4 ± 21.7	− 18.5 ± 16.1	0.001	0.128	0.770
Diastolic BP (mmHg)	91.8 ± 17.4	83.4 ± 16.2	− 8.4 ± 14.7	0.005	92.8 ± 14.9	85.9 ± 17.1	− 6.9 ± 9.6	0.001	0.826	0.570
Pulse pressure (mmHg)	83.7 ± 16.4	67.7 ± 16.9	16.0 ± 16.9	0.001	75.2 ± 13.3	62.4 ± 12.4	12.8 ± 13.6	0.001	0.035	0.183
Isolated SHT	15 (50%)				13 (46%)				0.799	

Data are expressed as mean and standard deviation

*BMI* body mass index, *BP* blood pressure, *SHT* systolic hypertension

*p* values by: ^1^Wilcoxon test for paired comparisons; ^2^ANOVA for continuous variables; ^3^univariable analyses with baseline BP (or PP) as co-variate; ^4^Chi-squared and Fisher’s tests for categorical variables

## Discussion

The analysis of the present multi-center study focused on the evaluation of AD in patients with RH undergoing RDN. Major findings were the following: (1) AD can be easily derived from 4-chamber cine views with high reproducibility. No additional CMR sequences are needed to obtain comprehensive information about the central vasculature through AD. (2) RDN significantly improved AD in patients with RH, regardless of the BP response to the intervention. (3) There was evidence of age-independent improvements in AD after RDN.

Catheter-based RDN has been shown to reduce BP and sympathetic activity by modulation of renal sympathetic nerve fibers in animals and also humans [19, 21, 33]. Several clinical studies observed reductions in BP following RDN in patients with RH, but the results of the sham controlled Symplicity HTN-3 trial questioned the utility of RDN to lower BP [10, 26]. Various factors might explain the considerable heterogeneity in the effects of RDN on BP. Newer studies aim to avoid the confounding effects of BP lowering drugs and the variation in adherence to medical treatment [27]. At this point, the results of the proof-of-concept trial SPYRAL HTN-OFF MED suggest efficacy of RDN on BP in the absence of antihypertensive medications [13, 28]. Modulation of the sympathetic nervous system by RDN has been associated with reductions in left ventricular mass, improvements in obstructive sleep apnea severity, and occurrence of arrhythmias [3, 29–32]. A pilot study also demonstrated improved compliance measured by PWV after RDN, suggesting that these parameters better reflect the risk of later cardiac events than the reduction in SBP [33]. Augmentation index has also been shown to be beneficially affected by RDN [34]. Some of these pleiotropic effects may be explained by the increased sodium excretion which has been reported after RDN [35].

Here, we used CMR-based AD to assess the effect of RDN on aortic compliance. RDN demonstrated a beneficial impact on vascular stiffness 6 month post-procedure. This effect was independent of the BP lowering efficacy of the procedure and more pronounced the lower the compliance was at baseline. Almost half of our patients (48%) had isolated systolic hypertension, which mainly reflects the high rigidity and the decreased elasticity of the large vessels in this cohort. However, significant improvements in AD after RDN could be observed regardless of the type of hypertension.

During the natural course of arterial aging, structural and mechanical changes of the vascular wall lead to loss in elasticity and reduced vascular compliance [36]. An inverse relation of AD with age has been shown in several studies and most of the lifetime reduction in AD occurs before the age of 50 [4, 5, 37]. It is important to mention that the mean age herein was 64.4 ± 9.6 years, suggestive of a small range in AD improvement only. The observed changes in AD were indeed age dependent, but present among all age groups. This finding is congruent with the previous studies proving that the age-related decrease in systemic arterial stiffness is partially reversible [38]. Several authors have shown that the distal parts of the aorta seem to age slower than the more proximal ones [5, 39]. The performed measurements of AD in the descending aorta are hence appropriate to reflect vascular age in an elderly cohort.

**Fig. 5:**
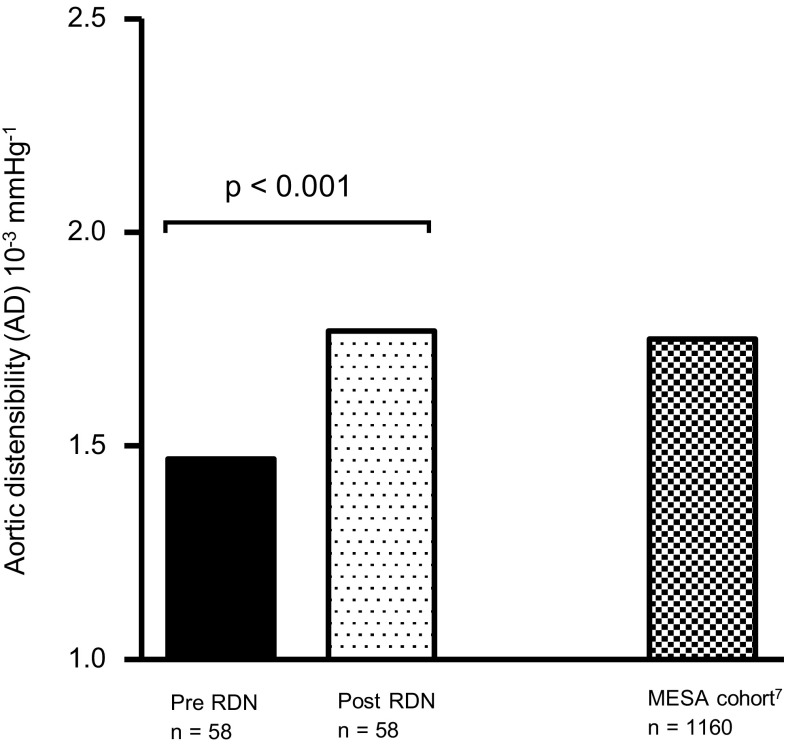
Evolution in median aortic distensibility (AD) from baseline to 6-month follow-up (left) in comparison with norm values reported in the MESA trial (right) [7]

The Multi-ethnic Study on Artherosclerosis (MESA) provided values of distensibility among 1160 healthy participants with a mean age of 60 ± 9 years [7]. Interestingly, the reported median descending AD in MESA of 1.75 × 10^−3^ mmHg^−1^ corresponds well with the median descending AD that our patients presented 6 months after RDN (1.77 × 10^−3^ mmHg^−1^) [7]. Figure 5 illustrates the evolution in our cohort from baseline to 6-month follow-up in comparison with the MESA collective. Hence, RDN improved the impaired AD in our cohort by about 30% and values appeared to return to levels obtained in an age-matched reference group 6 months after treatment. Importantly, our study supports the notion that renal denervation exerts clinically relevant effects beyond simple blood-pressure lowering, in this case pertaining to aortic vascular properties.

Longer term follow-up data will be important to better appreciate the apparent BP-independent effects of RDN and their impact on the cardiovascular risk profile of treated patients.

The identification of baseline predictors of response to RDN remains a central question of interest. We hypothesized that a reduction in SBP achieved by RDN was largest among patients with high AD and thus the lowest burden of aortic stiffness. However, this was not the case and response to RDN was independent of AD at baseline. Future trials will be needed to find out whether AD can ease the selection of patients with a likely response to RDN. Potentially, additional assessment of aortic wall calcification (evaluated by CT) already existing at baseline in combination with the assessment of vascular compliance by CMR will further increase the prediction of response to RDN [40].

## Limitations

The limitations of the Symplicity trials (NCT00664638, NCT00888433, and NCT01888315) have been discussed elsewhere [41]. The major limitations of our study include the non-randomized design and the fact that the study was not primarily designed to assess the effects of RDN on AD. Furthermore, the number of included patients was relatively small. However, we used CMR to explore effects on AD which represents a highly reproducible method compared to echocardiography, resulting in a considerable reduction in sample sizes of up to 90%. This has been underlined by the high ICC values of the present study despite the small cohort of patients. Measurements of PWV or any other marker of large vascular dysfunction reflecting vascular stiffness were not included in the study protocol, making a direct comparison of these parameters impossible. Office BP but not central BP was used to calculate AD. Central BP measured during MRI would have been very likely to provide more precise information on AD. The invasiveness though clearly limits the feasibility of these measurements; hence, we decided to rely on non-invasive brachial pressures. A limitation of this trial is the missing sham group. A placebo or Hawthorne effect can, therefore, not be fully excluded. Finally, the adherence to medication could not be confirmed by urine levels of antihypertensive drugs. Patients had specific instructions not to change antihypertensive therapy during the study period. Only one patient in our cohort reported to have increased his dose of diuretics. Medication changes may have effects on arterial stiffness in addition to RDN and may influence AD.

## Conclusion

Our results underline the direct neurohormonal influence of RDN on vascular tone and aortic stiffness and propose that CMR determined AD may be most suitable in the evaluation of aortic compliance in invasive BP therapy. Indeed, other parameters considered to integrate alterations of vascular compliance and arterial stiffness such as isolated systolic hypertension, augmentation index, and pulse wave velocity have been demonstrated to improve with RDN. By evaluating AD in the descending aorta, we present a simple, robust, and reproducible method to record changes in aortic stiffness. The present data illustrate that the effects of RDN are not limited by age and can improve compliance even in patients with a low-baseline compliance. A next step would be to include AD as part of an aortic routine analysis to further evaluate the prognostic value of this method.

## Electronic supplementary material

Below is the link to the electronic supplementary material.

Supplementary material 1 (DOCX 63 KB)

## Abbreviations

AD: Aortic distensibility
ANOVA: Analysis of variance
BP: Blood pressure
CMR: Cardiac magnetic resonance tomography
AD: Aortic distensibility
DBP: Diastolic blood pressure
ICC: Intra-class coefficient
ISH: Isolated systolic hypertension
LV: Left ventricle
LVEF: Left ventricular ejection fraction
LVH: Left ventricular hypertrophy
PP: Pulse pressure
PWV: Pulse wave velocity
RDN: Renal sympathetic denervation
RH: Resistant hypertension
RV: Right ventricle
SBP: Systolic blood pressure
SD: Standard deviation
SSFP: Steady-state free precession sequence
