# Supplementary material for: Renal sympathetic denervation restores aortic distensibility in patients with resistant hypertension: data from a multi-center trial
Source: Clin Res Cardiol. 2018 Mar 8;107(8):642–52. doi: 10.1007/s00392-018-1229-z (PMC6060801; doi:10.1007/s00392-018-1229-z)
Supplement: Supplementary file 1 — Supplementary material 1 (DOCX 63 KB) [file 392_2018_1229_MOESM1_ESM.docx]

**Supplementary material**

# Renal sympathetic denervation restores aortic distensibility in patients with resistant hypertension:

# Data from a multi-center trial

Lukas Stoiber^1^, Felix Mahfoud^2,3^, Seyedeh Mahsa Zamani^1^, Tomas Lapinskas^1,8^, Michael Böhm^2^, Sebastian Ewen^2^, Saarraaken Kulenthiran^2^, Markus P. Schlaich^4,5^, Murray D. Esler^5^,Tommy Hammer^6^, Knut Haakon Stensæth^6^, Burkert Pieske^1,7,9^, Stephan Dreysse^1^, Eckart Fleck^1^, Titus Kühne^9,10,11^, Marcus Kelm^9,10,11^, Philipp Stawowy^1^ and Sebastian Kelle^1,7,9^

^1^German Heart Center Berlin, Department of Internal Medicine/Cardiology, Berlin, Germany
^2^Division of Cardiology, Angiology and Intensive Internal Medicine, Department of Internal Medicine III, Saarland University, Homburg, Germany

^3^Institute for Medical Engineering and Science, Massachusetts Institute of Technology, Cambridge, MA, USA

^4^ Dobney Hypertension Centre, School of Medicine, University of Western Australia - Royal Perth Hospital Unit,

^5^Baker Heart and Diabetes Institute, Melbourne, Australia
^6^Department of Radiology and Nuclear Medicine, St. Olavs Hospital and Institute of Circulation and Medical Imaging, Norwegian University of Science and Technology, Trondheim, Norway

^7^Charité Campus Virchow Klinikum, Department of Internal Medicine/Cardiology, Berlin, Germany
^8^Department of Cardiology, Medical Academy, Lithuanian University of Health Sciences, Kaunas Lithuania 
^9^DZHK (German Center for Cardiovascular Research), Partner Site Berlin

^10^Department of Congenital Heart Disease and Pediatric Cardiology, German Heart Center Berlin, Berlin, Germany

^11^Institute for Computational and Imaging Science in Cardiovascular Medicine, Charité Berlin

**Correspondence**

Lukas Stoiber, MD
German Heart Center Berlin
Augustenburger Platz 1
13353 Berlin
Germany
phone: ++49 30 4593 2486
fax. ++49 30 4593 2500
E-mail: [stoiber@dhzb.de](mailto:stoiber@dhzb.de)

**Figure S1**

**Figure S2**

Inter-observer variability (A) Intra-observer variability (B)

- 1.96 SD

+ 1.96 SD

- 1.96 SD

Mean

+ 1.96 SD

Mean

**Figure S3**

**Figure S4**

**Figure S5**

**Table S1**

| **Parameter** | Pearson’s  correlation coefficients for baseline AD | **P-Value** | Pearson’s  correlation coefficients for Follow-up AD | **P-Value** | Pearson’s  correlation coefficients for change in AD | **P-Value** |
| --- | --- | --- | --- | --- | --- | --- |
| - Age | -0.317 | p = 0.015 | -0.497 | p < 0.001 | -0.227 | p = ns |
| - Male gender | 0.026 | p = ns | 0.081 | p = ns | 0.061 | p = ns |
| - BMI | 0.037 | p = ns | -0.09 | p = ns | -0.127 | p = ns |
| - Diabetes type 2 | -0.230 | p = ns | 0.012 | p = ns | 0.034 | p = ns |
| - No. of BP drugs | -0.100 | p = ns | -0.219 | p = ns | -0.137 | p = ns |
| - Isolated SHT | 0.011 | p = ns | -0.057 | p = ns | -0.070 | p = ns |

AD: aortic distensibility; BP: blood pressure; BMI: body mass index; No: number; ns: not significant; SHT: systolic hypertension

**Table S2**

| **Interobserver variability (A)**  **ICC values 95%-CI** | | **Intraobserver variability (B)**  **ICC values 95%-CI** | | |
| --- | --- | --- | --- | --- |
| 0.986 | 0.973 - 0.992 | 0.886 | 0.796 | 0.938 |
| 0.993* | 0.987 - 0.996 | 0.940* | 0.886 | 0.968 |

*Average values, ICC: intraclass-coefficient; CI: confidence interval. All data are presented as mean ± standard deviation.

**Table S3**

| **Parameter** | **All patients (n = 58)** |
| --- | --- |
| - No. of antihypertensive drugs at follow-up | 4.5 ± 1.7 |
| ACE inhibitors /ARBs | 51 (88%) |
| β-blockers | 46 (79%) |
| Calcium Channel blockers | 46 (79%) |
| Diuretics | 49 (84%) |
| Sympatholytics | 24 (41%) |
| Direct renin-Inhibitors | 18 (31%) |

Data is expressed as mean and standard deviation. No. = number; BMI = body mass index; SHT = systolic hypertension; BP = blood pressure; ACE = angiotensin converting enzyme; ARB = angiotensin receptor blocker
